# Injury to the palmar supporting structures of the fetlock alters limb stiffness and fetlock angle

**DOI:** 10.1111/evj.14409

**Published:** 2024-09-01

**Authors:** Katherine Hanousek, Andrew Fiske‐Jackson, Lauren O'Leary, Roger K. W. Smith

**Affiliations:** ^1^ Equine Referral Hospital Royal Veterinary College Hertfordshire UK

**Keywords:** fetlock conformation, horse, ligament, limb stiffness, superficial digital flexor tendon, suspensory ligament, tendon

## Abstract

**Background:**

In vivo measurement of limb stiffness and conformation provides a non‐invasive proxy assessment of superficial digital flexor tendon (SDFT) and suspensory ligament (SL) function. Here, we compared it in fore and hindlimbs and after injury.

**Objectives:**

To compare the limb stiffness and conformation in forelimbs and hindlimbs, changes with age, and following injury to the SDFT and SL.

**Study design:**

Retrospective cohort study.

**Methods:**

Limb stiffness was calculated using floor scales and an electrogoniometer taped to the dorsal fetlock. The fetlock angle and weight were simultaneously recorded five times with the limb weight‐bearing and when the opposite limb was picked up (increased load). Limb stiffness of both limbs was calculated from the gradient of the regression line of angle versus load. Fetlock angle when the weight was zero was extrapolated from the graph and used as a measure of conformation. Limb stiffness was measured in uninjured forelimbs (*n* = 42 limbs), hindlimbs (*n* = 19 limbs), forelimbs with SDFT injury (*n* = 18) and hindlimbs with SL injury (*n* = 5).

**Results:**

Limb stiffness correlated with weight in forelimbs as shown previously (*p* < 0.001) but also in hindlimbs (*p* = 0.006). When normalised to the horse's weight (503 kg, IQR 471.5–560), forelimb stiffness was significantly higher (22.3 [±4.5] × 10^−3^ degree^−1^) than for the hindlimb (16.4 [±4.0] × 10^−3^ degree^−1^; *p* < 0.001). While there were no significant differences between forelimb and hindlimb conformation in unaffected or SDFT injury, both limb stiffness and conformation was significantly greater in limbs with SL injury (*p* = 0.009 and *p* = 0.002, respectively).

**Main limitations:**

Small sample size, lack of clinical data including lameness and quantification of injuries.

**Conclusions:**

Injury to the forelimb SDFT does not alter limb stiffness or conformation in the long‐term, while hindlimb SL injury simultaneously increases limb stiffness and fetlock angle, suggesting an increase in SL length following injury.

## Zusammenfassung

**Hintergrund:**

Die in vivo Messung der Steifigkeit und Konformation der Gliedmaßen bietet eine nicht‐invasive Möglichkeit zur Beurteilung der Funktion der oberflächlichen Beugesehne (SDFT) und des Fesselträgers (SL). Diese wurde bei Vorder‐ und Hintergliedmaßen sowie nach einer Verletzung verglichen.

**Ziele:**

Der Vergleich der Steifigkeit und Konformation der Gliedmaßen bei Vorder‐ und Hintergliedmaßen, Veränderungen im Alter und nach Verletzungen der SDFT und SL.

**Studiendesign:**

Retrospektive Kohortenstudie.

**Methoden:**

Die Steifigkeit der Gliedmaßen wurde mithilfe von Bodenwaagen und einem an das dorsale Fesselgelenk geklebten Elektrogoniometer berechnet. Der Fesselwinkel und das Gewicht wurden gleichzeitig fünfmal erfasst, sowohl mit tragender Gliedmaße als auch bei Anheben der gegenüberliegenden Gliedmaße (erhöhte Belastung). Die Steifigkeit der Gliedmaßen beider Beine wurde aus der Steigung der Regressionslinie von Winkel zu Last berechnet. Der Fesselwinkel bei einem Gewicht von null wurde aus dem Diagramm extrapoliert und als Maß für die Konformation verwendet. Die Steifigkeit der Gliedmaßen wurde bei unverletzten Vordergliedmaßen (*n* = 42 Gliedmaßen), Hintergliedmaßen (*n* = 19 Gliedmaßen), Vordergliedmaßen mit SDFT‐Verletzung (*n* = 18) und Hintergliedmaßen mit SL‐Verletzung (*n* = 5) gemessen.

**Resultate:**

Die Steifigkeit der Gliedmaßen korrelierte mit dem Gewicht bei den Vordergliedmaßen, wie zuvor gezeigt (*p* < 0,001), aber auch bei den Hintergliedmaßen (*p* = 0,006). Nach Normierung auf das Gewicht des Pferdes (503 kg, IQR 471,5–560) war die Steifigkeit der Vordergliedmaßen signifikant höher (22,3 [±4,5] × 10^−3^ Grad‐1) als die der Hintergliedmaßen (16,4 [±4,0] × 10^−3^ Grad‐1; *p* < 0,001). Während es keine signifikanten Unterschiede zwischen der Konformation von Vorder‐ und Hintergliedmaßen bei unbeeinträchtigten oder SDFT‐verletzten Tieren gab, waren sowohl die Steifigkeit als auch die Konformation bei Gliedmaßen mit SL‐Verletzung signifikant größer (*p* = 0,009 bzw. *p* = 0,002).

**Wichtigste Einschränkungen:**

Kleine Stichprobengröße, Mangel an klinischen Daten, einschließlich Lahmheit und Quantifizierung der Verletzungen.

**Zusammenfassung:**

Eine Verletzung der oberflächlichen Beugesehne (SDFT) der Vordergliedmaße verändert die Steifigkeit oder Konformation der Gliedmaße langfristig nicht, während eine Verletzung des Fesselträgers (SL) der Hintergliedmaße gleichzeitig die Steifigkeit der Gliedmaße und den Fesselwinkel erhöht, was auf eine Verlängerung des Fesselträgers nach der Verletzung hindeutet.

## 1 INTRODUCTION

Injuries to the superficial digital flexor tendon (SDFT) and suspensory ligament (SL) are the most common soft tissue injuries in the forelimbs and hindlimbs, respectively, of athletic horses.^1^, ^2^, ^3^, ^4^ As primary supporters of the fetlock joint, the digital flexor tendons and SL are adapted to act as springs, reducing the metabolic cost of locomotion.^5^, ^6^ This spring function is achieved through the fetlock's unique capacity to significantly change the length of the limb, while muscle contraction in the proximal limb has relatively little impact on overall limb stiffness.^7^ The fore and hindlimbs have further adapted different kinematic roles in locomotion,^8^, ^9^ which suggests they may require different biomechanical properties, but the relative limb stiffness of the fore‐ and hindlimbs has not previously been investigated.

The flexor tendons appear to have limited adaptive response to loading following skeletal maturity, after which matrix synthesis has ceased and exercise causes microtrauma leading to matrix degeneration.^10^, ^11^ This degeneration is thought to predispose to exercise‐associated over‐strain injury through unclear mechanisms, which may be either biological or mechanical.^12^, ^13^ However, changes in tendon structural properties have not been consistently found with ageing in mature horses.^14^ One study identified a reduction in SDFT stiffness in mature horses compared with those under 24 months of age,^15^ while another study demonstrated a decrease in fetlock joint angles with age in immature horses, suggesting an increase in tendon stiffness as they reach maturity.^16^

An improved understanding of the biomechanical properties of the distal limb will provide further insight into the propensity for, and biomechanical response to, soft tissue injury. Mechanical studies in horses have shown that there is a linear correlation between load on the limb and fetlock angle when the limb is unloaded^7^ and this allows a measurement of limb stiffness (in kg/degree) to be calculated. Non‐invasive, in vivo measurement of limb stiffness has been demonstrated to be significantly correlated to in vitro SDFT stiffness after SDFT injury^17^ and the measurement of in vivo limb stiffness in a clinical setting using an electrogoniometer and floor scales has been validated and demonstrated to have excellent repeatability.^18^

The objectives of this study were to determine the differences in limb stiffness and fetlock conformation (predicted fetlock angle when the load on the limb is zero) between uninjured forelimbs and hindlimbs, and how this changes with age. The second objective was to understand how limb stiffness and conformation changes over time following injury to the forelimb SDFT and hindlimb SL.

## 2 MATERIALS AND METHODS

### 2.1 Study population

A convenience sample of limb stiffness data from the RVC was gathered retrospectively from clinical records of horses with unilateral forelimb SDFT and hindlimb SL injuries, diagnosed using a combination of clinical examination (swelling of the respective structure) and ultrasonographic evidence of injury of the structure as follows:

- Forelimb SDFT group (*n* = 18)—a lesion identified in the forelimb SDFT. Injuries to the SDFT were defined by swelling of the tendon, pain on palpation, and loss of fibre pattern and hypoechogenic lesions visible ultrasonographically.
- Hindlimb SL group (*n* = 5)—a unilateral lesion identified in the hindlimb proximal SL, the body or the branches of the SL. Injuries to the SL were defined by ultrasonographic evidence of enlargement, loss of fibre pattern and hypoechogenic lesions within the SL.

Horses were only included if pathology was identified ultrasonographically unilaterally, because of the need to express limb stiffness as a ratio to compare between cases because of the influence of weight.^18^ Horses were also excluded if multiple pathologies were identified in the affected limb.

**FIGURE 1:**
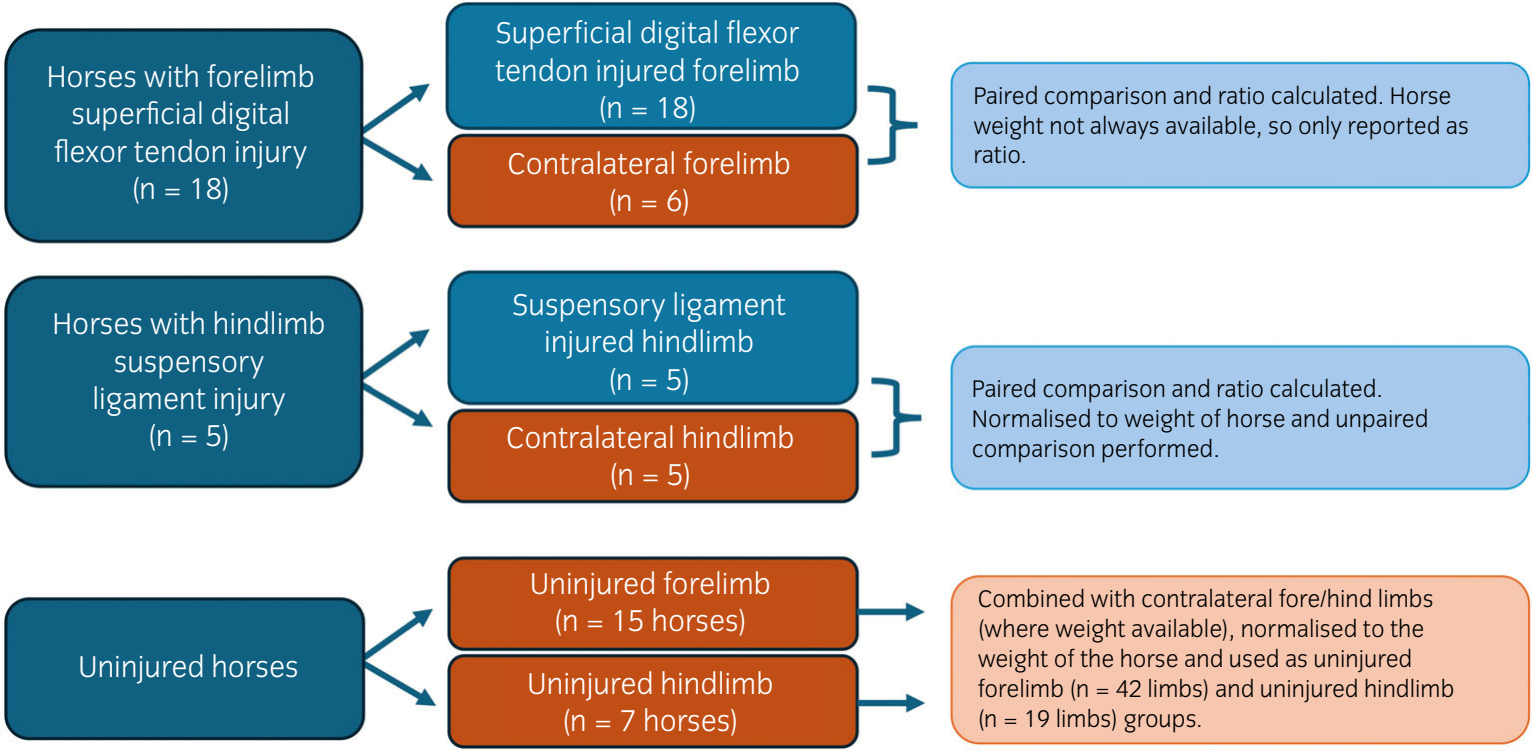
A flow chart depicting which limbs were used for which analyses.

The uninjured horse population comprised of horses owned by the Royal Veterinary College, and privately owned horses presenting to the RVC for problems unrelated to the measured limbs. All horses had no reported lameness, and no ultrasonographic evidence of soft tissue injury in the measured limbs. Historic limb stiffness measurements, obtained 10 years prior to the date of the study, were available for three of the university owned horses. Historic and current limb stiffness measurements were compared for these three horses, and the historic measurements were otherwise excluded from analysis. A flowchart depicting which limbs were used in which analysis is presented in Figure 1.

### 2.2 Limb stiffness measurement

All limb stiffness measurements were performed by the authors (KH, AFJ, RKS), who used a previously described technique,^18^ which involved the simultaneous measurement of fetlock angle and weight through the limb, while the contralateral limb is lifted and lowered in order to change load through the measured limb. A twin axis SG150B goniometer (Biometrics Ltd) was attached to the dorsal aspect of the pastern (short base plate) and third metacarpus or metatarsus (long base plate) with zinc oxide tape in order to measure fetlock angle. The horse stood on a custom‐made wooden box, which housed a 0–600 kg (±0.2)kg floor scales (My Scales), allowing the weight to be measured under one limb while the contralateral limb was lifted and lowered. In order to facilitate limb stiffness measurements horses were sedated with an intravenous dose of detomidine hydrochloride (0.005–0.02 mg/kg) and butorphanol (0.005–0.02 mg/kg). Simultaneous readings of both the fetlock angle and the weight were obtained five times with the contralateral limb lifted and with both limbs equally weight‐bearing, and the process was repeated for both fore‐ or both hindlimbs. The gradient of the regression line of angle versus weight was the limb stiffness.

Because limb stiffness has been shown to vary with the weight of the horse, limb stiffness data were normalised to the bodyweight of the horse in all uninjured horses and in all horses with SL injuries. Bodyweight was not available for 14 of the horses in the SDFT group and so, to control for the unknown bodyweights, the ratio of left and right limbs was calculated for each horse with an SDFT injury.

### 2.3 Fetlock conformation

The regression line of fetlock angle and weight was used to extrapolate the fetlock angle when the weight of the limb is 0 kg. This was used as a standardised measure of fetlock conformation (‘resting’ fetlock angle).

### 2.4 Data analysis

Data were analysed using R studio (R Studio). The identity of the horses was masked for analysis. Comparison was performed using paired limb stiffness measurements for each horse, or the ratio of paired injured and uninjured limbs. Normality of the data was confirmed with a Shapiro–Wilk test, and homogeneity of variances was confirmed with the Levene test. The relationship between limb stiffness and conformation, and these variables' relationship with the age of the horse and the duration of the injury were evaluated using a Pearson's product–moment correlation test (function ‘cor.test’ in the R ‘stats’ package). The relationship between limb stiffness and conformation of the injured versus contralateral, or left versus right, and front versus hindlimbs was evaluated using a paired t‐test (function ‘t.test’ in the R ‘stats’ package).

## 3 RESULTS

Details of the number of limbs measured in each group, the age and bodyweight of the horses can be found in Table 1. Limb stiffness and conformation data for uninjured forelimbs and hindlimbs, and for the hindlimb SL group was normally distributed (*p* > 0.05), and met the assumption of homogeneity of variance (*p* > 0.05) when normalised to the weight of the horse. All horses in the SDFT group had unilateral disruption of the tendon evident ultrasonographically in the mid metacarpal region of variable severity. Of the 18 horses with SDFT injuries, 12 were affecting the left forelimb and 6 were affecting the right forelimb. Horses with SL injuries had a variety of proximal SL, body, and branch injuries detailed in Table 2. All horses in the SL group were Warmbloods, the SDFT group was a mixture of Thoroughbreds (*n* = 12) and Warmbloods (*n* = 6). The uninjured group consisted of a mixture of Warmbloods (*n* = 7), Thoroughbreds (*n* = 3), and ponies (*n* = 5).

**TABLE 1:** The number of horses that underwent limb stiffness measurements, the number of individual limbs, the range and median age of the horses (years) and the median bodyweight (kg).

	Number of horses measured	Number of limbs measured	Age range of horses (years)	Median age of horses (years)	Median bodyweight (kilograms)
Uninjured forelimbs	21 (15 bilaterally uninjured)	42	3–35	14 (inter‐quartile range 10–21)	503 (inter‐quartile range 471.5–560)
Uninjured hindlimbs	12 (7 bilaterally uninjured)	19	6–35	15 (inter‐quartile range 13–24.5)	610 (inter‐quartile range 573–620)
Superficial digital flexor tendon injury	18	18	3–24	9 (inter‐quartile range 8–11)	560 (inter‐quartile range 531–588)
Suspensory ligament injury	5	5	12–21	18 (inter‐quartile range 18–20.75)	604 (inter‐quartile range 557.75–625)
Total	45	100	3–35	12 (inter‐quartile range 8.25–18)	586 (inter‐quartile range 497.74–620)

Horses were grouped based on limb injury. The total number of horses is less than the sum of all groups combined as some horses contributed to multiple groups.

**TABLE 2:** The clinical presentation, chronicity and ultrasonographic findings of horses in the injured suspensory ligament group.

Horse	Injured limb	Presentation	Ultrasonographic findings	Chronicity (weeks)
1	Left	Lameness with swelling and pain on palpation of the suspensory ligament branches.	Medial and lateral suspensory ligament branch injury, core lesion in the suspensory ligament body. Remodelling of both medial and lateral proximal sesamoid bones.	4
2	Left	Lameness, blocked to the deep branch of the lateral plantar nerve.	Enlargement (approximately 10% compared with contralateral limb) of the proximal suspensory ligament with several regions of hypoechogenicity. Osseous remodelling of the third metatarsal bone of the affected limb.	16
3	Right	Lameness	Core lesion of the proximal suspensory ligament with marked enlarged compared with the contralateral limb.	22
4	Right	Lameness, blocked to the deep branch of the lateral plantar nerve.	Small hypoechogenic dorsal border lesion in the proximal suspensory ligament, with mild entheseopathy at the site of the lesion. No enlargement of the ligament.	34
5	Right	Lameness with swelling and pain on palpation of the suspensory ligament branches.	Enlargement and periligamentous fibrosis of both medial and lateral suspensory ligament branches, with loss of fibre pattern of the lateral branch.	227

### 3.1 Limb stiffness in uninjured limbs

**FIGURE 2:**
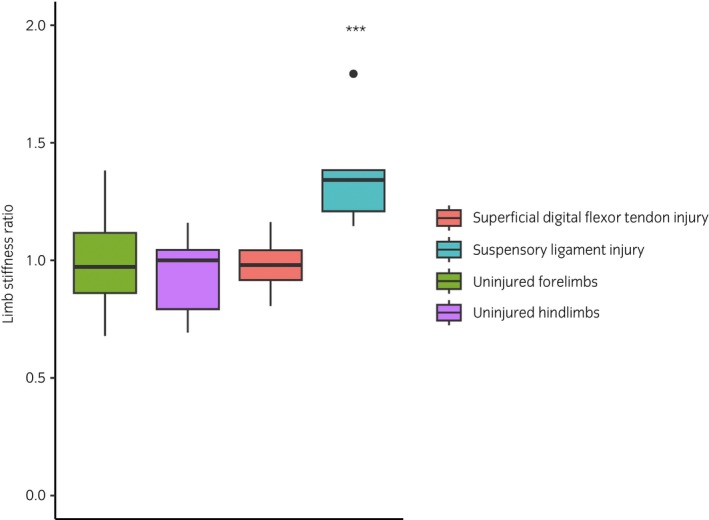
The ratio of limb stiffness between the injured and contralateral limbs of horses with superficial digital flexor tendon (*n* = 18) injuries and suspensory ligament (*n* = 5) injuries, and the left and right limbs of uninjured forelimbs (*n* = 15) and hindlimbs (*n* = 7). In the suspensory ligament group, the injured limbs were significantly stiffer (mean = 0.018 degree^−1^, ±0.003) than their paired contralateral limb (mean = 0.014 degree^−1^, ±0.003, *t* = 3.63, *p* < 0.001).

The analysis of uninjured limbs included paired measurements from uninjured horses, and the contralateral limbs from injured horses. Where paired values were available from bilaterally uninjured horses, there was no significant difference between the limb stiffness of the left and right forelimbs (*n* = 15 pairs) or hindlimbs (*n* = 7 pairs) (Figure 2). There was no significant difference between the limb stiffness of paired uninjured limbs and uninjured contralateral limbs for forelimbs or hindlimbs.

**FIGURE 3:**
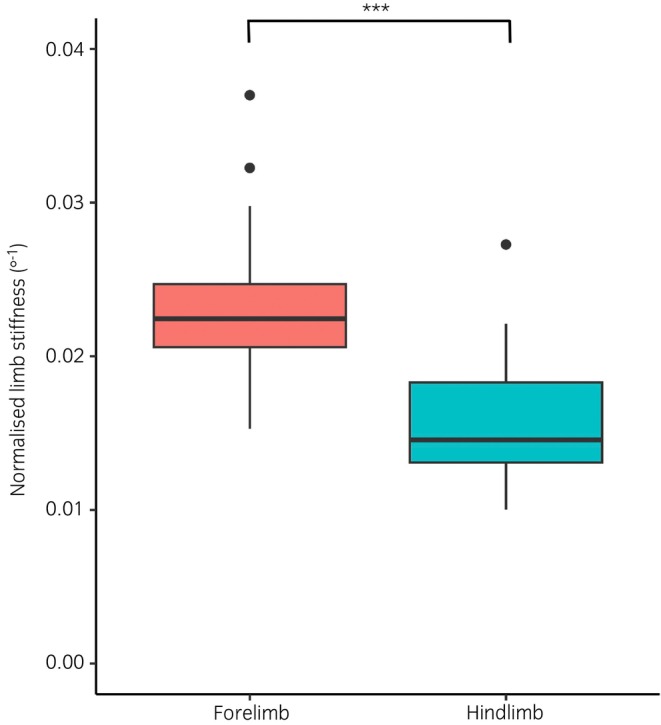
Limb stiffness in uninjured forelimbs (*n* = 15) and hindlimbs (*n* = 7), normalised to the weight of the horse (*p* < 0.001).

**FIGURE 4:**
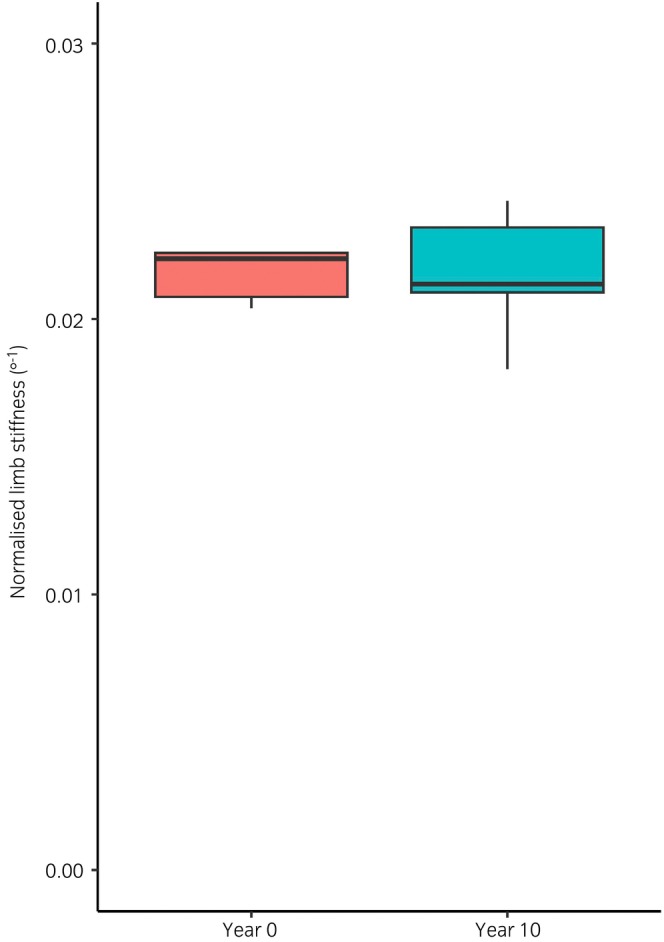
The limb stiffness of three horses, repeated 10 years apart, normalised to the weight of the horse on the day of measurement (*p* > 0.9).

As shown previously,^18^ limb stiffness was positively correlated with bodyweight both in forelimbs (*r* = 0.82, *p* < 0.001), and in hindlimbs (*r* = 0.61, *p* < 0.001). When normalised to the weight of the horse, uninjured forelimb limb stiffness was significantly higher (0.023 degree^−1^ ±0.005) than uninjured hindlimb limb stiffness (0.016 degree^−1^ ±0.004, *t* = 9.122, *p* < 0.001), as shown in Figure 3. The age of the horse was not correlated with limb stiffness in either the forelimbs (*r* = 0.12) or the hindlimbs (*r* = 0.26). Longitudinal limb stiffness measurements, repeated 10 years apart, were also available for the forelimbs of three horses and are shown in Figure 4. This showed no significant difference between the limb stiffness in year 0 compared with year 10 (*t* = 0.021, *p* > 0.9; Figure 4), the average coefficient of variation between the two time points was 4.93%.

### 3.2 Fetlock conformation in uninjured limbs

There was no significant difference between the conformation of paired uninjured limbs, and uninjured contralateral limbs for forelimbs or hindlimbs. Where paired values were available from bilaterally uninjured horses, there was no significant difference between the conformation of the left and right forelimbs (*n* = 15 pairs) or hindlimbs (*n* = 7 pairs). Fetlock conformation was not significantly different between forelimbs (163°, ±25°) and hindlimbs (161°, ±14°). Conformation was not significantly correlated with the limb stiffness (forelimb *r* = 0.15, hindlimb *r* = 0.25), the age of the horse (forelimb *r* = 0.03, hindlimb *r* = 0.30), or the weight of the horse (forelimb *r* = 0.001, hindlimb *r* = 0.08).

### 3.3 Limb stiffness and fetlock conformation in horses with forelimb SDFT injuries

The duration of the SDFT injuries at the time of measurement ranged from 1 to 180 weeks (mean 39 weeks). There was no difference between the injured and the paired contralateral limbs in limb stiffness or conformation. There was no correlation between the duration of the injury and the limb stiffness (*r* = −0.04), or conformation (*r* = −0.07) of the injured limb. The ratio of injured to uninjured limbs in the SDFT group was 0.97 (±0.11), as shown in Figure 2.

### 3.4 Limb stiffness and fetlock conformation in horses with hindlimb SL injuries

The duration of the SL injuries at time of measurement ranged from 4 to 281 weeks (mean 90 weeks). The injured limbs were significantly stiffer (mean = 0.018 degree^−1^, ±0.003) than their paired contralateral limb (mean = 0.014 degree^−1^, ±0.003, *t* = 3.63, *p* < 0.001). The ratio of injured to uninjured limbs for the whole group was 1.27 (±0.22) as shown in Figure 2. Fetlock conformation was also significantly reduced in the injured limbs (mean = 152°), compared with the contralateral limbs (mean = 165°, *t* = 3.47, *p* = 0.002). There was no correlation between the duration of the injury and the injured limb stiffness (*r* = 0.08) or conformation, although there was a trend for the fetlock conformation to increase with duration (*r* = 0.63, *p* = 0.09). A post‐hoc power calculation revealed a sample size of 18 horses would be required to identify a significant correlation between injured fetlock conformation and duration of injury.

## 4 DISCUSSION

The key findings of this study are that while there is no change in two fetlock mechanical parameters following SDFT injury in the forelimb, these are altered following injury to the hindlimb SL; limb stiffness is increased alongside fetlock conformation (greater fetlock drop). Although Dakin et al.^17^ identified a correlation between limb stiffness and SDFT stiffness, it is important to emphasise that this study only measures limb stiffness and not tendon stiffness. Inferences about changes in stiffness of individual tendons and ligaments are based on comparing limbs with specific injuries to uninjured limbs. However, the change in fetlock parameters after SL injury suggests an increase in the overall length of the SL as a different consequence of injury to the SDFT. All measurements in this study were obtained from sedated horses, as in the validation study performed by Jacklin et al.^18^ Sedation was used because it was hypothesised that voluntary muscle contraction would be less likely than in unsedated horses. However, Wilson and McGuigan demonstrated that the linear relationship between limb force and fetlock joint angle was minimally influenced by digital flexor muscle activation in vitro or as a function of gait in vivo.^7^

### 4.1 Uninjured limbs

In uninjured limbs, limb stiffness is significantly greater in the forelimb compared with the hindlimb, while fetlock conformation is the same, irrespective of age and weight and limb stiffness. This may reflect the differing roles of the forelimbs and hindlimbs in quadrupedal locomotion. The horse's centre of mass is located closer to the forelimbs than hindlimbs, resulting in greater load on the forelimbs. The forelimbs also provide energetic efficiency through their spring‐like action and contribute to deceleration, while the hindlimbs contribute more to propulsion.^6^, ^9^, ^19^ The peak ground reaction forces have been shown to be greater in the forelimbs than the hindlimbs at both the walk and trot.^8^, ^9^, ^20^, ^21^ By applying Hooke's law,^22^ we can deduce that in order to achieve a similar deflection (fetlock angle) with increased load, the limb stiffness must be higher in the fore‐ than hindlimbs to optimise spring‐like properties. These differences in fore‐ and hindlimb stiffness are reflected by an equivalent reduced elastic modulus of the hindlimb compared with the forelimb.^23^, ^24^

The difference in limb stiffness may also have relevance to the risk of injury. Both SDFT and SL injuries are common in the forelimb, while SL injuries are more common than SDFT injuries in the hindlimb,^1^, ^25^, ^26^ where they have been associated with straight hocks and collapsed fetlocks in sports horses.^27^ A possible explanation for this is that with a straighter hock a greater proportion of the load will be taken by the SL for a given fetlock extension.

This study is not able to determine which component, or components, of the supporting structures of the fetlock are responsible for the difference between forelimbs and hindlimbs. Dakin et al.^17^ found a positive correlation between in vivo limb stiffness measurements and ex vivo SDFT stiffness, but did not explore how this relates to the deep digital flexor tendon (DDFT) or SL. Payne et al.^28^ found both the SDFT and the flexor digitorum lateralis of the DDFT to be less stiff in the hindlimb than in the forelimb, which is consistent with the results of this study and suggests that both the SDFT and DDFT are implicated in the reduced hindlimb stiffness. Whether this difference is reflected in the fore‐ and hindlimb SL is yet to be established.

### 4.2 Age and limb stiffness

This study demonstrated no significant change in limb stiffness with age. However, the horses used in the study were all mature. The biggest changes in SDFT composition and mechanical properties occur during growth where tendon maturity is estimated to occur between 2 and 3 years of age.^10^, ^15^, ^29^, ^30^ Following maturity, the digital flexor tendons are considered to change little other than the accumulation of microdamage which is thought to predispose the tendon to injury.^12^ In vivo kinematic studies have used high speed photography to capture the change in fetlock angle in a moving horse, which is a similar measure of limb stiffness to that which is this study, and showed that 2‐year‐old Thoroughbred racehorses had quicker rates of fetlock dorsiflexion than older horses, which were not significantly different to one another.^14^ Another study investigated fetlock joint angles in three age‐groups of horses—immature (up to 1.5 years of age), mature (3–8 years of age) and aged (10–19 years of age).^16^ They demonstrated a decrease in maximal extension during growth in immature horses, but the maximal fetlock joint extension remained constant through to aged horses, consistent with our study which used horses aged between 3 and 35 years. In further support of a lack of change in limb stiffness was particularly relevant data generated by this study, although only three horses, which further confirmed no change in limb stiffness over a 10‐year period with very little difference between the two time‐points. One of the limitations of the study is that, given its retrospective nature, only a relatively small number of horses were analysed. Further work is required to investigate this, ideally using longitudinal samples from groups of working and non‐working horses.

### 4.3 Limb stiffness following SDFT injury

Dakin et al.^17^ previously described a reduction in limb stiffness following injury to the SDFT, which increased to match the contralateral limb between 2 and 7 months (7 and 30 weeks) post injury. With no longer term follow‐up on these cases, Dakin et al.^17^ posed the question of whether the stiffness of the injured limb would remain equivalent to the contralateral limb or become stiffer beyond 7 months. If the stiffness of the injured limb were to continue to increase, exceeding that of the contralateral limb, Dakin et al.^17^ suggested this would lead to changes in the energy storing efficiency of the limb and could predispose the limb to re‐injury or reduce performance. With up to 180 weeks follow‐up, this study has demonstrated that there is no long‐term alteration in limb stiffness or conformation following injury to the SDFT. This sheds light on the remarkable ability of injured SDFT to match the contralateral (normal) limb's stiffness during healing which makes perfect sense for a structure that plays a key role for elastic energy storage and therefore performance. This is indeed consistent with the findings of O'Meara et al.^31^ who showed that racing performance is not affected following recovery from injury to the SDFT. However, as the injured area of tendon has been shown to have increased stiffness due to fibrosis,^32^ the adjacent non‐injured areas of tendon must be under increased strain, providing a mechanism for subsequent increased susceptibility to injury, which is usually seen at an adjacent or remote site to the original injury, despite no overall change in limb stiffness.

### 4.4 Limb stiffness and conformation following SL injury

Limited work has been carried out on the biomechanics of the equine SL following injury. The equine SL is unique in that the collagen fibres are interspersed with muscle and adipose tissue,^33^ which makes it difficult to compare with ligament healing studied in other species.

This study demonstrated that SL injury caused an increase in fetlock extension, which remained the case regardless of the duration of the injury (4 to 280 weeks). Increased fetlock angle is a previously reported feature of SL disease,^33^, ^34^ although, to the authors' knowledge, it has not been quantified previously. This increase in fetlock extension could arise through a decrease in limb stiffness. However, in contrast, we identified that this increased fetlock extension was associated with an increase in limb stiffness. This suggests that there is a permanent increase in the length of the ligament following injury to the SL, in contrast to after SDFT injury. Further work is necessary to determine the mechanism of this elongation, and also determine if this change is progressive over time. A limitation of this study is that, due to low case numbers, all SL injuries were grouped together. Further research should aim to evaluate injuries to the proximal SL, the body and the branches independently. Further to this, this study lacked any quantification of injury to the SL or any clinical parameters, such as lameness.

Limb stiffness and fetlock conformation measurements essentially quantify is appreciable clinically. In that way, it has some value for the diagnosis of suspensory disease although such a diagnosis relies on many other aspects including clinical examination, and diagnostic analgesia and imaging. In addition, there is some overlap in the limb stiffness between normal and injured horses and hence will only be supportive as a diagnostic feature for those horses where the values exceed that of the normal horses. It will be of greater value for the monitoring the progression of clinical disease as then each horse acts as its only control and reduces the inter‐horse variability.

The clinical use of these measurements remains to be fully evaluated. However, it does enable an in vivo quantification of limb mechanics which we believe is of relevance to the management of these injuries. It has demonstrated that the clinical identification of fetlock collapse post injury can arise through two different mechanisms, namely decreased stiffness, such as those demonstrated by Dakin et al.^17^ in acute SDFT injuries, or increased length of the healing structure with increased stiffness (chronic suspensory disease). Fetlock collapse has been associated with a poor prognosis^35^ and so these values could have prognostic value. Similarly, any reductions in these parameters should prompt consideration of adding methods of fetlock support to the rehabilitation programme.^12^

One limitation of this study was that it was limited to SDFT injuries in the forelimb and SL injuries in the hindlimbs. It would, of course, be also interesting in future work to evaluate the effects of the corresponding injuries of the converse limbs and for injuries to other (secondary) supporting structures, such as the deep digital flexor tendon, but these injuries were much less numerous in our data set, preventing meaningful analysis.

In summary, we have identified different limb stiffnesses between fore‐ and hindlimbs and changes in limb stiffness and conformation associated with different injuries. These data may be helpful, not only to understand the aetiopathogenesis of overstrain injury of the supporting structures of the distal limb in the horse, but also the different processes in the healing of SDFT versus SL injury. Furthermore, this technique may also provide additional, mechanical‐based, monitoring of healing in clinical cases.

## FUNDING INFORMATION

Not applicable.

## CONFLICT OF INTEREST STATEMENT

The authors declare no conflicts of interest.

## AUTHOR CONTRIBUTIONS

**Katherine Hanousek:** Conceptualization; investigation; writing – original draft; methodology; writing – review and editing; data curation. **Andrew Fiske‐Jackson:** Writing – review and editing; data curation. **Lauren O'Leary:** Data curation. **Roger K. W. Smith:** Writing – review and editing; writing – original draft; supervision; data curation; methodology; conceptualization; investigation.

## DATA INTEGRITY STATEMENT

Katherine Hanousek had full access to all the data in the study and takes responsibility for the integrity of the data and the accuracy of data analysis.

## ETHICAL ANIMAL RESEARCH

Ethical approval for this research was granted by the Royal Veterinary College Ethical Approval Board (URN 2023 2204‐2).

## INFORMED CONSENT

Owners gave consent for their animals' inclusion in the study.

### PEER REVIEW

The peer review history for this article is available at https://www.webofscience.com/api/gateway/wos/peer‐review/10.1111/evj.14409.

## Data Availability

The data that support the findings of this study are openly available in figshare at http://doi.org/10.6084/m9.figshare.25526302.
